# Diagnosis of somatoform disorders in primary care: diagnostic agreement, predictors, and comaprisons with depression and anxiety

**DOI:** 10.1186/s12888-018-1940-3

**Published:** 2018-11-12

**Authors:** Katharina Piontek, Meike C. Shedden-Mora, Maria Gladigau, Amina Kuby, Bernd Löwe

**Affiliations:** 1 Institute for Medical Psychology, University Medicine Greifswald, Greifswald, Germany; 2 Department of Psychosomatic Medicine and Psychotherapy, University Medical Center Hamburg-Eppendorf, Hamburg, Germany

**Keywords:** Somatoform disorders, Depressive disorder, Anxiety disorder, Primary care Diagnostic agreement, Detection rate, Structured clinical interview

## Abstract

**Background:**

To investigate (a) the diagnostic agreement between diagnoses of somatoform disorders, depressive and anxiety disorders obtained from a structured clinical interview and diagnoses reported from primary care physicians (PCPs) and (b) to identify patient and PCP-related predictors for the diagnostic agreement regarding the presence of a somatoform disorder.

**Methods:**

Data from a cross-sectional study comprising 112 primary care patients at high risk for somatoform disorders were analyzed. Diagnoses according to International Classification of Diseases, 10th revision (ICD-10) for somatoform, depressive and anxiety disorders were obtained from the Composite International Diagnostic Interview (CIDI) and compared with the diagnoses of the patients’ PCPs documented in their medical records. Using multiple regression analyses, predictors for the PCPs’ diagnosis of a somatoform disorder were analyzed.

**Results:**

The agreement between PCP diagnoses and CIDI diagnoses was 32.3% for somatoform disorders, 48.0% for depressive disorders and 25.0% for anxiety disorders. Multiple regression analyses revealed the likelihood of being diagnosed with a somatoform disorder by PCP increased with somatic symptom severity (OR = 1.22, 95% CI 1.03–1.44). Regarding PCP-related characteristics, a specialization in internal medicine (OR = 5.95, 95% CI 1.70–20.80) and working in a solo practice (OR = 2.92, 95% CI 1.02–8.38) increased the likelihood that patients were diagnosed with a somatoform disorder.

**Conclusions:**

The present results indicate that the process of diagnosing somatoform disorders in primary care needs to be improved. Findings further underline the necessity to implement appropriate strategies to improve early detection of patients.

**Trial registration:**

ISRCTN ISRCTN55870770. Registered 22 October 2014. Retrospectively registered.

## Background

The burden of disease due to non-specific, functional and somatoform disorders is high and the presence of recurrent or persistent medically unexplained symptoms is associated with impaired physical and mental quality of life, increased utilization of healthcare services and the development of comorbidities like depressive and anxiety disorders [1, 2]. Available data regarding the prevalence of somatoform disorders are inconsistent and vary considerably in dependence on the underlying study population and the diagnostic criteria applied in the single studies (e.g. [3–6]). A recent meta-analysis of 32 studies investigated the prevalence of somatoform disorders and medically unexplained symptoms in primary care patients using both strict diagnostic criteria according to clinical assessments (*International Classification of Diseases; ICD* [7], *Diagnostic and Statistical Manual of Mental Disorders; DSM* [8]) and standardized questionnaires [9]. That study revealed a high heterogeneity of the primary studies and substantial differences in the prevalence rates in the diagnoses of somatoform disorders according to ICD or DSM criteria with prevalence rates of 34.8% and 26.2%, respectively. Further, in 40% to 49% of the primary care patients, at least one medically unexplained symptom was detected using questionnaires. These numbers underline that somatoform disorders pose a highly relevant public health problem, but existing literature suggests that the process of diagnosing somatoform disorders is challenging and that the different diagnostic criteria may have a substantial impact on the detection rate. The low detection rate in primary care is one key problem in the management of somatoform disorders [6]. It has been demonstrated that only 33% to 60% of the patients are correctly diagnosed by their primary care physician (PCP) and referred to a specialist for further treatment [4]. This is particularly alarming as the primary care practice serves as the patient’s first point of entry into the health care system and access to mental health services. Furthermore, according to current guidelines for non-specific, functional, and somatoform bodily complaints [1], management of somatoform disorders is recommended within a stepped care model according to the course of disease, in collaboration with other physicians and therapists and coordinated by the PCP, thereby emphasizing the high responsibility of the PCP in the diagnostic and therapeutic procedure [10].

Several barriers have been identified with respect to the diagnosis of somatoform disorders in primary care settings [11]. Present data indicate that a lack of specific training, a lack of experience and a lack of reliable diagnostic tools may hinder the diagnosis of somatoform disorders [11]. It has been further demonstrated that the application of the diagnostic criteria seem to be problematic for many PCPs and existing classification systems have been described as being difficult to use, impractical, not distinct, overlapping or too restrictive [11]. Besides these conceptual barriers, both patient and PCP-related characteristics might contribute to a correct diagnosis, and it is of importance to identify relevant predictors of a correct PCP diagnosis of a somatoform disorder to improve early detection of the disease.

The aims of the present study are, first, to investigate the level of agreement between diagnoses of somatoform disorders and its comorbidities depression and anxiety disorder obtained from a structured clinical interview and diagnoses reported from PCPs and, second, to identify patient and PCP-related predictors of PCPs’ diagnoses of a somatoform disorder.

## Methods

### Sample recruitment

Data were collected within the project “Network Somatoform and Functional disorders” (*Sofu-Net*), a sub-project of the Hamburg Network for Mental Health *psychenet* [12, 13]. The study is registered at ISRCTN (ISRCTN55870770). *Sofu-Net* aimed to improve early detection of patients with somatoform disorders in primary care and to refer patients more quickly into effective treatment [14].

Between September and December 2012, all patients at least 18 years old attending the participating primary care practices were asked to take part in a screening regarding bodily complaints and well-being. Patients with severe physical illness, cognitive impairment or insufficient German language skills were excluded from the study. Patients with a positive screening result were asked to participate in a telephone interview within 4 weeks after the screening. All patients gave informed written consent. The study protocol is consistent with the principles of the Declaration of Helsinki and was approved by the ethics committee Medical Chamber Hamburg, Germany.

### Measures

The self-administered screening questionnaire included data on the patients’ age, gender, marital status, and school education. The Patient-Health-Questionnaire (PHQ) [15] was used to screen for somatoform disorders (PHQ-15), depression (PHQ-9) and anxiety (Generalized Anxiety Disorder Assessment; GAD-7) [16]. The PHQ-15 comprises of 15 items assessing somatic symptoms and their severity within the last 4 weeks. The 3-point Likert scale ranges from *not bothered* (0) to *bothered a lot* (2). The PHQ-9 comprises nine items assessing depressive symptoms and their severity within the last 2 weeks. The 3-Point Likert Scale ranges from *not at all* (0) to *nearly every day* (2). The GAD-7 comprises of seven items assessing anxiety-related symptoms within the last 2 weeks. The 3-Point Likert Scale ranges from *not at all* (0) to *nearly every day* (2). On all three scales, cutoff-values of 5, 10 and 15 represent mild, moderate and severe symptom levels, respectively [15]. Patients were considered screening positive if one the following conditions was fulfilled: (a) PHQ-15 ≥ 15, (b) PHQ-15 ≥ 10 and GAD-7 or PHQ-9 ≥ 10. Further, patients were asked how often they talk about psychosocial complaints and private problems with their PCP using the following question: ‘Do you talk about psychological complaints and private problems with your primary care physician?’ Patients responded on a 6*-*point Likert Scale ranging from *never* (1) to *always* (6).

The telephone interview encompassed the sections for somatoform disorders, depressive and anxiety disorders of the Composite International Interview (CIDI) [17]. The CIDI assesses mental disorders according to the criteria of the *International Classification of Diseases, 10th revision* (ICD-10) [7] and the criteria of the *DSM-IV Diagnostic and Statistical Manual of Mental Disorders, 4th edition* (DSM-IV) [8]. For the present analyses, diagnostic codes according to ICD-10 were analyzed. ICD-10 diagnoses obtained from the CIDI were considered as reference standard. CIDI interviews were conducted by research assistants who were extensively trained and supervised. Patients fulfilling the diagnostic criteria within the last 6 months were classified as having a current somatoform disorder, depressive or anxiety disorder. The category of somatoform disorders included somatization disorder (F45.0), undifferentiated somatoform disorder (F45.1), and persistent pain disorder (F45.4). Within a decision tree, it was assessed for each reported complaint whether it was medically unexplained. Mild, moderate and severe depressive episodes as well as dysthymia were summarized in the category depressive disorders (F32-F34). Anxiety disorders included agoraphobia, social phobia, specific phobias (e.g. animal phobias), phobic anxiety disorder, panic disorder, and generalized anxiety disorder (F40-F41).

#### PCP data form

The PCPs completed a form assessing their patients’ medical history based on the data in their medical records. This form consisted of 6 sections encompassing the following topics: (1) reason for the patients’ consultation (using ICD-10 codes), (2) present somatic diseases (list of 26 diseases and an additional open question, e.g. cardiovascular, endocrine, neurological, lung and autoimmune diseases), (3) present mental disorders (list of 13 disorders and an additional open question, e.g. dementia, alcohol, drug and medication dependence, schizophrenia, depression, anxiety disorder, somatoform disorder), (4) somatic explanation of the patients’ bodily symptoms from the PCPs’ perspective, (5) recommendation of psychological treatment by PCP, and (6) intake of medication (antidepressants, analgesics, benzodiazepines, and antipsychotic drugs). The form was completed by PCPs for all screening-positive patients when the screening procedure had been finished.

#### Data from treating physicians

Using a questionnaire, the following characteristics of the participating PCPs were assessed: age, gender, specialization, training in psychosomatic basic care, years in general practice, and type of practice.

### Data analyses

Data are reported as means (standard deviation) for continuous variables and as numbers (percentages) for categorical variables. Group comparisons were performed using t-tests (mean) for continuous variables and Pearson χ^2^-tests for categorical variables. Tests were considered statistically significant at a two-sided *p*-value < 0.05. Kappa-statistics were calculated to determine the diagnostic agreement between diagnoses of somatoform disorders, depressive and anxiety disorders obtained from the CIDI and diagnoses obtained from PCPs. The following agreement levels were considered: discrete (0–0.20), regular (0.21–0.40), moderate (0.41–0.60), substantial (0.61–0.80), almost perfect (0.81–1.00) [18].

Two multiple logistic regression analyses were conducted to identify predictors for a PCP diagnosis of a somatoform disorder. The diagnoses obtained from CIDI were used as reference standard. The first model included the following patient-related predictors: age, gender, marital status, school education, comorbid depressive disorder, comorbid anxiety disorder, discussion of psychosocial problems with PCP, somatic symptom severity, and the number of PCP visits within the last 6 months. The second model included the following PCP-related predictors: age, gender, specialization, years in general practice, training in psychosomatic basic care, and type of practice.

Data analyses were conducted using STATA 13.0 (Stata Corporation, College Station, TX, USA).

## Results

### Characteristics of the study population

**Fig. 1:**
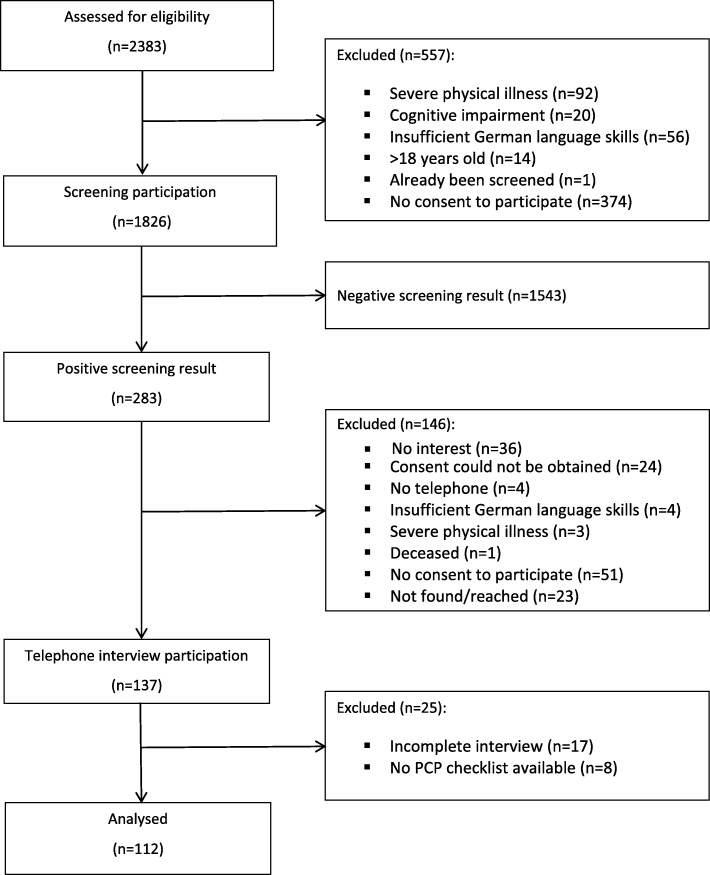
Flow-chart according to sample recruitment

From 1826 screening participants, 283 (15.5%) were screened positive for somatoform disorders, depression and anxiety. From those screening positive, 137 (48.4%) patients participated in the telephone interview. After the exclusion of patients with incomplete interviews and missing data, 112 cases were available for the present analyses (Fig. 1).

Among the study population, the majority of patients was female. Data showed that patients with and without somatoform disorders did not differ in their sociodemographic and clinical characteristics (Table 1).

**Table 1:** Characteristics of the study population according to the presence of a somatoform disorder as diagnosed using CIDI

	Total	With somatoform disorder	Without somatoform disorder
	*n* = 112	*n* = 97 (86.0%)	*n* = 15 (14.0%)
Female sex, n (%)	88 (78.6%)	79 (81.4%)	9 (60.0%)
Age (years), M (SD)	47.1 (15.9)	45.5 (15.2)	57.5 (17.1)
School education, n (%)			
< 10 years	28 (25.5)	25 (26.0)	3 (21.4)
10 years	35 (31.8)	29 (30.2)	6 (42.9)
> 10 years	47 (42.7)	42 (43.8)	5 (35.7)
Marital status, n (%)			
Married	42 (37.8)	35 (36.5)	7 (46.7)
Unmarried	38 (34.2)	35 (36.5)	3 (20.0)
Separated, divorced, widowed	31 (27.9)	26 (27.1)	5 (33.3)
PHQ-15 score, M (SD)	14.6 (3.3)	14.9 (3.4)	13.2 (2.6)
Age (years) at the onset of somatoform symptoms, M (SD)	19.3 (13.7)	19.3 (13.7)	n/a
Comorbid depressive disorder (CIDI) within the last 6 months, n (%)	50 (44.6)	43 (44.3)	7 (46.7)
Comorbid anxiety disorder (CIDI) within the last 6 months, n (%)	48 (42.9)	43 (44.3)	5 (33.3)
Currently in psychotherapy, n (%)	24 (22.0)	21 (22.3)	3 (20.0)
PCP visits within the past 6 months, M (SD)	6.1 (4.8)	6.0 (4.4)	6.6 (6.7)
Discussion of psychosocial distress with PCP, n (%)			
Never, seldom/rarely, sometimes	69 (61.6)	59 (60.8)	10 (66.7)
Often, very often, always	43 (38.4)	38 (39.2)	5 (33.3)

*CIDI* Composite International diagnostic interview

*PHQ-15* Patient Health Questionnaire

*PCP* Primary care physician

Within the study population, 65 (58.0%) patients were diagnosed with a current somatoform disorder according to CIDI and 29 (25.9%) patients were diagnosed with a current somatoform disorder according to their PCP. The rates of comorbidity with current depressive or anxiety disorders according to CIDI were 43.1 and 47.7%, respectively, while 26.6% of the patients presented both disorders (data not shown).

### Characteristics of the participating PCPs

The characteristics of the participating PCPs are illustrated in Table 2. PCPs were on average 49.3 years old, and 53.7% were female. Most PCPs had a specialization in general medicine (58.5%) and 85.4% had completed training in psychosomatic basic care. Most frequently, PCPs worked in a group practice (75.6%) and had worked in general practice for on average 10.5 years.

**Table 2:** Characteristics of PCPs (*n* = 41)

Age (years), M (SD)	49.3 (7.7)
Female sex, n (%)	22 (53.7)
Specialization, n (%)	
General medicine	24 (58.5)
Internal medicine	12 (29.3)
Other specialization	5 (12.2)
Training in psychosomatic basic care, n (%)	35 (85.4)
Type of practice, n (%)	
Solo practice	10 (24.4)
Group practice	31 (75.6)
Years in general practice, M (SD)	10.5 (5.6)

### Diagnostic agreement

The results of the analyses regarding the diagnostic agreement are displayed in Table 3. Using the CIDI diagnosis as gold standard, data analyses revealed that PCPs diagnosed 21 (32.3%) patients with a somatoform disorder, while they did not reach agreement with the CIDI diagnosis in 44 (67.7%) patients. PCPs classified 8 (17.0%) patients as having a somatoform disorder although a negative result was obtained using CIDI. Diagnostic agreement of not having a somatoform disorder according to CIDI or PCPs was found for 39 (83.0%) patients. Kappa value was 0.14 (*P* < .05), indicating a significant discrete diagnostic agreement.

**Table 3:** Diagnostic agreement between PCP ratings and CIDI results (*n* = 112)

		CIDI	
		n (%) yes	n (%) no
Somatoform disorder			
PCP rating	n (%) yes	21 (32.3)	8 (17.0)
	n (%) no	44 (67.7)	39 (83.0)
Depressive disorder			
PCP rating	n (%) yes	24 (48.0)	14 (22.6)
	n (%) no	26 (52.0)	48 (77.4)
Anxiety disorder			
PCP rating	n (%) yes	12 (25.0)	5 (7.8)
	n (%) no	36 (75.0)	59 (92.2)

Regarding depressive disorders, PCPs diagnosed 24 (48.0%) patients, while they did not reach agreement with the CIDI diagnosis in 26 (52.0%) patients. In contrast, PCPs classified 14 (22.6%) patients as having a depressive disorder although a negative result was obtained using CIDI. Diagnostic agreement of not having a depressive disorder according to CIDI or PCPs was found for 48 (77.4%) patients. Kappa value was 0.26 (*P* < 0.01), indicating a significant regular diagnostic agreement.

Regarding anxiety disorders, PCPs diagnosed 12 (25.0%) of patients, while they did not reach agreement with the CIDI diagnosis in 36 (75.0%) patients. In contrast, PCPs classified 5 (7.8%) patients as having an anxiety disorder although a negative result was obtained using CIDI. Diagnostic agreement of not having an anxiety disorder according to CIDI or PCPs was found for 59 (92.2%) patients. Kappa value was 0.19 (*P* < 0.01), indicating a significant discrete diagnostic agreement.

### Patient and PCP-related predictors for the diagnosis of a somatoform disorder by PCPs

Regarding patient-related characteristics, multiple regression analysis revealed that with every point increase in PHQ-15 mean score, the chance of being diagnosed with a somatoform disorder increased by 1.2 (OR = 1.22; 95% CI 1.03–1.44) (Table 4).

**Table 4:** Patient-related predictors for PCPs’ diagnosis of a somatoform disorder (*n* = 85)

Patient-related characteristics	OR (95% CI)	*P*-value
Age	1.00 (0.97; 1.05)	0.75
Female gender	0.42 (0.10; 1.67)	0.22
Marital status, married	1.52 (0.75; 3.10)	0.25
At least 10 years of schooling	1.45 (0.71;2.96)	0.31
Comorbid depressive disorder	1.70 (0.52; 5.55)	0.38
Comorbid anxiety disorder	0.92 (0.20; 4.32)	0.92
Discussion of psychosocial distress	1.90 (0.60; 6.00)	0.27
Somatic symptom severity (PHQ-15)	1.22 (1.03; 1.44)	0.04
Number of PCP visits within the last 6 months	0.94 (0.80; 1.10)	0.44

Results of multiple logistic regression analysis

*PHQ-15* Patient Health Questionnaire

*PCP* Primary care physician

Regarding PCP-related characteristics, multiple regression analysis revealed that a specialization in internal medicine was associated with a 6-fold higher chance that patients were diagnosed with a somatoform disorder by their PCP (OR = 5.95; 95% CI 1.70–20.80) (Table 5). Further, working in a solo practice was associated with a 3-fold higher chance of patients being diagnosed with a somatoform disorder (OR = 2.92; 95% CI 1.02–8.38). With respect to the PCPs’ age, younger age was associated with a higher chance of patients being correctly diagnosed with a somatoform disorder (OR = 0.90; 95% CI 0.81–1.00). This effect was borderline significant (*p* = 0.06). In the single models, all predictors explained 11.8 and 17.2% of the model variance, respectively.

**Table 5:** Physician-related predictors for PCPs’ diagnosis of a somatoform disorder (*n* = 99)

Physician-related characteristics	OR (95% CI)	*P*-value
Age	0.90 (0.81; 1.00)	0.06
Female sex	2.26(0.74; 6.85)	0.15
Specialization internal medicine	5.95 (1.70; 20.80)	<.01
Years in general practice	1.06 (0.93; 1.21)	0.38
Training in psychosomatic basic care	0.58 (0.15; 2.96)	0.51
Solo practice	2.92 (1.02; 8.38)	<.05

Results of multiple logistic regression analysis

## Discussion

In the present population of primary care patients at high risk for somatoform disorders, the diagnostic agreement between PCPs’ diagnoses and diagnoses obtained from a structured clinical interview was highest for depressive disorders, followed by somatoform disorders and anxiety disorders. Our analyses further revealed that somatic symptom severity was a relevant patient-related predictor for PCPs’ diagnosis of a somatoform disorder, while a specialization in internal medicine and working in a solo practice were relevant physician-related predictors for PCPs’ diagnosis of a somatoform disorder.

We showed that PCPs identified 32.3% of the patients with a somatoform disorder using the CIDI diagnosis as reference. This finding is in line with data from previous studies which revealed agreement rates ranging from 18% to 56%, depending on the investigated populations and the methods applied in the single studies [19–22]. This variety may reflect the difficulties in the terminology and conceptualization of somatoform type disorders which have often been discussed in previous research [11]. Regarding conceptual barriers in the process of diagnosing somatoform disorders, existing classification systems have been described by PCPs as being difficult to use, as providing little information about the illness, or as too restrictive [11].

In the present study, all PCPs participating in *Sofu-Net* had attended network meetings and quality circles which were an inherent part of the network providing information about diagnostic approaches and management of somatoform disorders. Moreover, participating PCPs were prompted to use the PHQ as screening tool for early detection and contemporary referral to psychotherapeutic co-treatment. We therefore suppose that participation in *Sofu-Net* has promoted the PCPs’ awareness for patients with somatoform complaints and their diagnostic knowledge and skills. Further, among the participating PCPs, 85% had a training in psychosomatic basis care, suggesting that these PCPs may be particular interested in the field of psychosomatic care which might has enhanced the detection of patients with somatoform complaints. In addition, PCPs were aware of the patients’ positive screening result, giving advice that somatoform complaints were present in their patients. Nevertheless, using CIDI as reference, a large part of the patients with somatoform as disorders remained unrecognized by their PCPs. However, it is of importance to note that among all patients who had been “overlooked” by their PCPs (67.7%), PCPs stated for 54.6% of these patients that they do not consider the patients’ complaints as medically explained and therefore suspect the presence of a somatoform disorder. This finding suggests that PCPs were aware of somatization in their patients, although not having labelled their patients as having a somatoform disorder. A recent qualitative study by our group among PCPs investigated the process of coding somatoform disorders in primary care [23]. In that study, PCPs reported that coding is done for reimbursement purposes, that they use other information in their personal documentation, for example about the patients’ psychosocial background or potential causes about the presented symptoms, and that they do not necessarily need to document a confirmed diagnosis for treatment. Moreover, PCPs reported that they restrain their coding to protect patients from stigma trough certain diagnoses or other negative consequences. Inaccurate coding was further described to arise from uncertainties regarding the diagnostic criteria and that finding the definite diagnosis is seen as the responsibilities of psychiatric specialists [23]. Although we have not investigated these processes in the present study, it might be assumed that the described factors were of relevance among the PCPs in the present study and have contributed to the relatively low detection rate.

In the present study, the diagnostic agreement was highest for depressive disorders (48%). It has been argued that PCPs are better acquainted with depression than with other mental disorders and may therefore have a greater ability to detect depressive disorders [24]. Regarding the presence of an anxiety disorder, the diagnostic agreement was only 25.0%. When interpreting this result, it needs to be considered that the diagnostic classification of anxiety disorders in the present study encompassed conditions of differential severity (i.e. panic disorder, animal phobia). It is possible that PCPs did not to diagnose light cases, given that symptoms might not have been apparent or might not have been associated with severe impairments. In turn, patients might have not disclosed their symptoms when feeling only slightly impaired.

We demonstrated that somatic symptom severity was a relevant predictor for a diagnosis of a somatoform disorder by PCPs, while the patient’s sociodemographic characteristics had no impact on the detection rate. Possibly, an increasing symptom severity presumably has prompted patients to describe their bodily complaints more often and striking to their PCPs, thereby facilitating the detection of a somatoform disorder.

We further showed that a specialization in internal medicine and working in a solo practice were relevant physician-related predictors for a diagnosis of a somatoform disorder. This result is in line with findings from a study demonstrating that physicians who saw themselves as more effective in dealing with somatoform symptoms were more likely to be working in a solo practice [25]. Data from that study further showed that PCPs working in solo practices and those who were in the same practice for 5 years felt most comfortable in dealing with patients with somatoform disorders. It might be assumed that these PCPs had a better chance to establish a close relationship with their patients and had more time to explore their patients’ complaints and psychosocial background facilitating the diagnostic process. With regard to the PCPs’ education, we found that PCPs with training in internal medicine were more successful in diagnosing somatoform disorders compared to PCPs with training in general medicine, suggesting that training in internal medicine encompasses knowledge and skills facilitating the process of diagnosing somatoform disorders. It might be further possible that training in internal medicine makes PCPs more confident of declaring symptoms as medically explained, and thus diagnosing somatoform disorders. Taken together, the present findings underline that structural circumstances are essential for improving early detection and care for patients with somatoform disorders.

Strengths of the present study encompass the well characterized population of primary care patients at high risk for somatoform disorders as identified using established screening instruments and the structured diagnostic procedure using CIDI interviews. Our analyses are further based on different data sources including patient self-report, diagnoses obtained from CIDI interviews, and a PCP questionnaire, thereby integrating different perspectives on somatoform disorders diagnoses. Limitations may arise from the low response rate in the telephone interview, which potentially has introduced a selection bias. However, screening-positive patients who participated in the telephone interview did not differ from those who dropped out with regard to age, gender and PHQ-scores. Another weakness of the present study is the lack of data from screening-negative patients, which may has led to a misclassification bias underrepresenting the rate of patients with somatization. On the other hand, the PHQ has been demonstrated to be a well-validated measure for detecting and monitoring somatization, depression, and anxiety [15]. Nevertheless, as data on screening-negative patients are lacking, no data are available regarding PCPs diagnostic assessment of these patients.

## Conclusions

Our data demonstrate that the process of diagnosing somatoform disorders in primary care remains challenging. We showed that the diagnostic agreement between diagnoses obtained from CIDI and diagnoses obtained from PCPs was low, but there is reason to assume that PCPs were aware of somatization in their patients without labelling them as having a somatoform disorder. Even with the release of the DSM-5, conceptual and practical problems of the previous classification remain unresolved. However, our data indicate that structural circumstances are crucial in the diagnostic process, as PCPs training in internal medicine and working in a solo practice were associated with a higher chance of detecting a somatoform disorder. As structural circumstances are changeable, more research is necessary to assess and overcome situational barriers. Our data further suggest that there might be particular knowledge and skills taught during the education in internal medicine helping PCPs to detect patients with somatoform disorders. Likewise demonstrated in previous studies, the restricted time during consultations may hinder PCPs to build a strong doctor-patient relationship and to explore the psychosocial background of their patients, thereby impeding the correct diagnosis. Thus, continuity of care, as provided potentially stronger in solo practices, might facilitate the diagnosis of somatoform disorders in primary care. Furthermore, collaborative networks of PCPs and psychotherapists like *Sofu-Net* may contribute to the improvement of care in somatoform type disorders.

## Abbreviations

CI: Confidence interval
CIDI: Composite International Interview
DSM: Diagnostic and Statistical Manual of Mental Disorders
GAD: Generalized Anxiety Disorder Assessment
ICD: International Classification of Diseases
OR: Odds Ratio
PCP: Primary care physician
PHQ: Patient-Health-Questionnaire

## References

[1] Schaefert R, Hausteiner-Wiehle C, Hauser W, Ronel J, Herrmann M, Henningsen P (2012). Non-specific, functional, and somatoform bodily complaints. Dtsch Arztebl Int.

[2] Shedden-Mora M, Lau K, Kuby A, Gross B, Gladigau M, Fabisch A, Loewe B (2015). Improving health Care for Patients with somatoform and functional disorders: a collaborative stepped care network (Sofu-net). Psychiatr Prax.

[3] Loewe B, Spitzer RL, Williams JB, Mussell M, Schellberg D, Kroenke K (2008). Depression, anxiety and somatization in primary care: syndrome overlap and functional impairment. Gen Hosp Psychiatry.

[4] Steinbrecher N, Koerber S, Frieser D, Hiller W (2011). The prevalence of medically unexplained symptoms in primary care. Psychosomatics.

[5] de Waal MW, Arnold IA, Eekhof JA, van Hemert AM (2004). Somatoform disorders in general practice: prevalence, functional impairment and comorbidity with anxiety and depressive disorders. Br J Psychiatry.

[6] Fink P, Sorensen L, Engberg M, Holm M, Munk-Jorgensen P (1999). Somatization in primary care. Prevalence, health care utilization, and general practitioner recognition. Psychosomatics.

[7] World Health Organization (1992). The ICD-10 classification of mental and behavioural disorders: clinical description and diagnostic guidelines.

[8] American Psychiatric Association (2000). Diagnostic and statistical manual of mental disorders (4th ed., text rev.).

[9] Haller H, Cramer H, Lauche R, Gobos G (2015). Somatoform disorders and medically unexplained symptoms in primary care-a systematic review and meta-analysis of prevalence. Dtsch Arztebl Int.

[10] Hanel G, Henningsen P, Herzog W, Sauer N, Schaefert R, Szecsenyi J, Löwe B (2009). Depression, anxiety, and somatoform dirsorders: vague or distinct categories in primary care? Results from a large cross-sectional study. J Psychosom Res.

[11] Murray AM, Toussaint A, Althaus A, Lowe B (2016). The challenge of diagnosing non-specific, functional, and somatoform disorders: a systematic review of barriers to diagnosis in primary care. J Psychosom Res.

[12] Haerter M, Brandes A, Hillebrandt B, Lambert M (2015). psychenet - Das Hamburger Netz psychische Gesundheit [psychenet - The Hamburg Network for Mental Health]. Psychiatr Prax.

[13] Shedden-Mora MC, Gross B, Lau K, Gumz A, Wegscheider K, Loewe B (2016). Collaborative stepped care for somatoform disorders: a pre-post-intervention study in primary care. J Psychosom Res.

[14] Loewe B, Piontek K, Daubmann A, Harter M, Wegscheider K, Koenig HH, Shedden-Mora M (2017). Effectiveness of a stepped, collaborative, and coordinated health care network for somatoform disorders (Sofu-net): a controlled cluster cohort study. Psychosom Med.

[15] Kroenke K, Spitzer RL, Williams JB, Loewe B (2010). The patient health questionnaire somatic, anxiety, and depressive symptom scales: a systematic review. Gen Hosp Psychiatry.

[16] Spitzer RL, Kroenke K, Williams JB, Loewe B (2006). A brief measure for assessing generalized anxiety disorder: the GAD-7. Arch Intern Med.

[17] Kessler RC, Ustun TB (2004). The world mental health (WMH) survey initiative version of the World Health Organization (WHO) composite international diagnostic interview (CIDI). Int J Methods Psychiatr Res.

[18] Landis JR, Koch GG (1977). The measurement of observer agreement for categorical data. Biometrics.

[19] Pols RG, Battersby MW (2008). Coordinated care in the management of patients with unexplained physical symptoms: depression is a key issue. Med J Aust.

[20] Norton J, de Roquefeuil G, David M, Boulenger JP, Ritchie K, Mann A (2009). Prevalence of psychiatric disorders in French general practice using the patient health questionnaire: comparison with GP case-recognition and psychotropic medication prescription. Encéphale.

[21] Pilowsky I (1967). Dimensions of hypochondriasis. Br J Psychiatry.

[22] H G, Henningsen P, Herzog W, Sauer N, Schaefert R, Szecsenyi J, Lowe B (2009). Depression, anxiety, and somatoform disorders: vague or distinct categories in primary care? Results from a large cross-sectional study. J Psychosom Res.

[23] Pohontsch NJ, Zimmermann T, Jonas C, Lehmann M, Lowe B, Scherer M (2018). Coding of medically unexplained symptoms and somatoform disorders by general practitioners - an exploratory focus group study. BMC Fam Pract.

[24] Nuyen J, Volkers AC, Verhaak PF, Schellevis FG, Groenewegen PP, van den Bos GA (2005). Accuracy of diagnosing depression in primary care: the impact of chronic somatic and psychiatric co-morbidity. Psychol Med.

[25] Hartz AJ, Noyes R, Bentler SE, Damiano PC, Willard JC, Momany ET (2000). Unexplained symptoms in primary care: perspectives of doctors and patients. Gen Hosp Psychiatry.

